# RUNX3 regulates vimentin expression *via* miR-30a during epithelial–mesenchymal transition in gastric cancer cells

**DOI:** 10.1111/jcmm.12209

**Published:** 2014-01-22

**Authors:** Zhifang Liu, Long Chen, Xinchao Zhang, Xia Xu, Huaixin Xing, Yingjie Zhang, Wenjuan Li, Han Yu, Jiping Zeng, Jihui Jia

**Affiliations:** a Department of Biochemistry and Molecular Biology, School of Medicine, Shandong UniversityJinan, China; b Department of Anesthesiology, Shandong Cancer HospitalJinan, China; c Department of Radiation Oncology, Shandong Cancer HospitalJinan, China; d Department of Microbiology, Key Laboratory for Experimental Teratology of Chinese Ministry of Education, School of Medicine, Shandong UniversityJinan, China

**Keywords:** Gastric cancer, epithelial–mesenchymal transition, Runt-related transcription factor 3, miR-30a, vimentin

## Abstract

Runt-related transcription factor 3 (RUNX3) is a putative tumour suppressor *via* regulating the expression of a series of target genes. Clinical studies demonstrated that loss of RUNX3 expression is associated with gastric cancer progression and poor prognosis, but the underlying mechanism is not entirely clear. Accumulating evidence shows that the epithelial–mesenchymal transition (EMT) plays an important role in cancer relapse and metastasis. Therefore, we addressed whether RUNX3 has a role in the EMT in gastric cancer. Knockdown of RUNX3 promoted cell invasion and increased the protein expression of the mesenchymal marker vimentin in human gastric cancer cells. Overexpression of RUNX3 suppressed cell invasion and decreased the protein expression of vimentin in the cells and inhibited gastric cancer cells colonization in nude mice. Furthermore, overexpression of RUNX3 increased the expression of microRNA-30a (miR-30a), and miR-30a directly targeted the 3′ untranslated region of vimentin and decreased its protein level. miR-30a inhibitor abrogated RUNX3-mediated inhibition of cell invasion and downregulation of vimentin. Thus, RUNX3 suppressed gastric cancer cell invasion and vimentin expression by activating miR-30a. In gastric cancer patients, levels of RUNX3 were positively correlated with miR-30a and negatively associated with the levels of vimentin. Collectively, our data suggest a novel molecular mechanism for the tumour suppressor activity of RUNX3. Effective therapy targeting the RUNX3 pathway may help control gastric cancer cell invasion and metastasis by inhibiting the EMT.

## Introduction

Gastric cancer is one of the most common malignant tumours worldwide and ranks second in terms of global cancer-related death 1,2. The most common cause of the death from gastric cancer is invasion and metastasis of the tumour 3. Malignant tumour invasion and metastasis is a complex biological process, including the isolation of cancer cells from the primary lesion, invasion into the surrounding tissues, penetration of the basement membrane into blood vessels and lymphatic vessels, oozing from blood and lymphatic vessels, and reformation of metastatic nodules and then proliferation to new metastases 3–5. In the metastatic cascade, local invasion is considered an initial and essential step leading to distant metastasis 5,6. In this process, the epithelial–mesenchymal transition (EMT) was thought to play a critical role.

The EMT is a complex biological process whereby immotile, polarized epithelial cells are converted to cells with a motile mesenchymal cell phenotype, which enhances migratory capacity, invasiveness and resistance to apoptosis, thus contributing to cancer progression 7–10. The EMT programme is regulated by some transcriptional factors, including the zinc finger proteins Snail1, Slug and the basic helix–loop–helix factors Twist1/2 and Six, and the TGF-β and Wnt/β-catenin signalling pathways. These transcriptional factors or signalling pathways repress the expression of the epithelial marker E-cadherin and induce the expression of the mesenchymal marker vimentin 11–16.

Human runt-related transcription factor (RUNX) genes are homologues to *Drosophila Runt* 17 and *Lozenge* 18 genes and contain highly conserved sequences encoding the ‘runt domain’, which mediates the binding of RUNX protein to specific DNA sequences and causes transcriptional activation or repression of the downstream target genes 19,20. The human RUNX family has three members: RUNX1/AML1, RUNX2/CBFA1 and RUNX3/PEBP2aC/AML2. All three members share similar structures, but have different functions 21–23. Runt-related transcription factor 3 (RUNX3) is involved in the development of the gastrointestinal tract and neurogenesis during embryonic development. Recently, inactivation or loss of RUNX3 was found closely associated with tumourigenesis and tumour progression 24. RUNX3 is involved in transforming growth factor β (TGF-β)–induced growth inhibition and apoptosis. On TGF-β stimulation, RUNX3 cooperates with SMADs to directly up-regulate P21 and Bim, which inhibits cell growth and increases cell apoptosis respectively 25–27. RUNX3 deficiency caused a precancerous state in the gastric epithelia of mice 28. Recently, Lin *et al*. 20 showed that loss of RUNX3 expression enhanced the Akt1-mediated signalling pathway and increased tumourigenesis in human gastric cancer. Besides, RUNX3 expression was significantly reduced in clinical tissue samples of peritoneal metastasis in gastric cancer as compared with normal gastric mucosa or primary tumours 29. In addition, RUNX3 overexpression inhibited gastric cancer angiogenesis by decreasing the expression of VEGF and suppressed gastric cancer metastasis 30. Chang *et al*. 31 showed that RUNX3 could directly bind to the promoter of *Claudin-1* and inhibit tumourigenesis and metastasis in gastric epithelial cells. As well, RUNX3 suppressed gastric cancer metastasis by inactivating MMP9 *via* up-regulating TIMP-1 32.

Here, we investigated whether RUNX3 regulates the EMT in gastric cancer cells. We examined the effect of increased or decreased RUNX3 expression on the invasion potential of human gastric cancer cells and the expression of the EMT molecules vimentin and E-cadherin. Our data provide a novel mechanism for RUNX3-mediated suppression of gastric cancer invasion and metastasis.

## Materials and methods

### Patients

We obtained tumour specimens and surrounding normal tissue from 55 patients with primary gastric cancer who underwent gastrectomy at the Cancer Hospital of Shandong Province in 2012–2013. Samples were stored at −80°C. We collected data on patient age, sex and tumour histology, differentiation status, size (diameter), invasiveness, and regional and distant metastases at the time of surgery (pathologic tumour-node-metastasis classification). Detailed patient and disease characteristics are documented in Table 1. The study was approved by the ethics committee of School of Medicine, Shandong University.

**Table 1:** Patient and tumour characteristics, RUNX3 and vimentin protein expression in gastric cancer specimens

No.	Patient and tumour characteristics			Protein expression in cancer specimens‡		
	Age (year)/sex	Size (cm)	pTNM*	Histol/dif†	RUNX3	Vimentin
1	79/M	5.5	T4N2M0	MDA	0.56	0.91
2	56/M	5.5	T4N1M0	MDA	0.55	1.02
3	66/M	9.0	T4N3M0	MucinousA	0.11	3.85
4	64/F	3.5	T4N2M0	LDA	0.31	3.66
5	68/M	7.0	T4N3M1	MDA	1.11	1.22
6	51/M	3.0	T3N2M0	LDA	0.96	–
7	50/F	4.0	T4N2M0	LDA	0.08	4.83
8	58/M	2.0	T4N1M0	MDA	0.15	4.32
9	72/M	2.5	T4N3M0	LDA	1.23	0.23
10	63/M	5.5	T4N3M0	LDA	0.14	3.37
11	49/F	3.0	T4N1M0	LDA	–	4.51
12	70/F	4.0	T4N1M1	MDA	0.12	3.25
13	62/M	5.0	T4N1M0	MDA	1.95	0.52
14	62/M	6.0	T4N2M0	LDA	0.31	1.52
15	74/M	6.0	T3N0M0	MDA	1.45	–
16	38/F	8.0	T4N1M0	LDA	0.16	4.02
17	58/F	4.0	T4N3M1	LDA	1.56	–
18	54/M	7.0	T4N0M0	MucinousA	1.08	3.68
19	61/M	12	T4N1M0	LDA	0.25	3.58
20	49/M	3.0	T4N0M0	LDA	1.92	1.02
21	40/M	6.0	T4N1M0	MDA	0.59	–
22	61/M	5.0	T4N0M0	LDA	0.62	1.15
23	37/M	6.0	T4N3M0	LDA	0.23	4.22
24	59/M	5.0	T4N3M0	LDA	0.45	4.78
25	63/M	4.0	T4N1M0	MDA	0.87	–
26	50/F	4.0	T3N2M0	LDA	3.4	–
27	62/F	3.5	T3N0M0	LDA	0.96	0.02
28	72/M	4.1	T3N1M0	LDA	4.86	0.26
29	59/M	2.5	T4N0M0	MDA	1.24	0.62
30	65/M	6.0	T4N0M0	LDA	3.42	0.14
31	56/F	5.4	T4N2M0	LDA	1.43	1.33
32	54/M	4.0	T2N0M0	LDA	2.66	0.87
33	52/F	9.0	T4N1M0	MDA	2.65	–
34	46/F	2.0	T4N1M0	LDA	1.21	0.44
35	59/M	6.2	T3N0M0	LDA	4.46	–
36	68/M	7.0	T4N1M0	MucinousA	0.48	0.95
37	64/M	5.5	T4N1M0	LDA	4.36	0.64
38	45/M	10	T4N2M0	LDA	3.02	1.56
39	38/M	2.5	T3N2M0	LDA	1.03	4.72
40	52/M	6.5	T4N3M1	LDA	1.62	3.42
41	69/M	7.5	T4N3M1	LDA	1.79	3.08
42	75/M	9.5	T4N0MO	MDA	1.59	0.16
43	73/M	5.0	T4N1M0	MDA	1.73	0.75
44	50/M	4.0	T4N3M0	LDA	1.76	4.53
45	77/M	1.5	T4N2M0	LDA	2.11	1.57
46	52/M	1.5	T4N3M0	LDA	0.85	5.14
47	37/F	13	T4N3M1	LDA	0.61	4.54
48	66/F	3.5	T4N2M0	LDA	1.15	5.14
49	69/M	7.5	T4N3M1	LDA	1.37	5.21
50	40/F	5.0	T4N1M0	MucinousA	0.19	1.49
51	80/M	11	T4N3M1	MDA	0.55	5.12
52	52/F	9.0	T4N2M0	LDA	1.13	2.06
53	70/M	8.5	T4N3M1	LDA	0.78	5.13
54	73/M	6.0	T4N2M0	LDA	0.48	2.38
55	69/F	4.5	T4N1M0	MDA	0.48	1.12

* Pathologic tumour-node metastasis.

† Histology/Differentiation status: LDA, Low differentiated adenocarcinoma; MDA, Moderately differentiated adenocarcinoma; MucinousA, Mucinous adenocarcinoma.

‡ Semi-quantitative analyses of Runx3 and vimentin protein levels in cancer specimens using western blot and relative abundances of each protein were calculated based on band density values and normalized with β-actin.-: undetectable; RUNX3, runt-related transcription factor 3.

### Cell lines and reagents

Human gastric adenocarcinoma cell lines AGS, BGC-823, GES-1 and SGC-7901 (purchased from Cancer Institute Beijing, Beijing, China, 2008) and KATOIII cells (purchased from CLS-Cell Line Service, Eppelheim, Germany, 2008) free of mycoplasma (Checked using PCR, 2009) were used in this study. Cells were cultured at 37°C, 95% air, 5% CO_2_ in RPMI 1640 medium (Invitrogen, Carlsbad, CA, USA) containing 10% foetal bovine serum (FBS),100 U/ml penicillin and 2 mmol/l L-glutamine. Chemical modified Stealth small interfering RNAs (siRNAs) targeting RUNX3 and control siRNA were from Invitrogen. The sequence for the RUNX3 siRNA1, RUNX3 siRNA2 and control siRNA were 5′-AGUUCUCGUCAUUGCCUGCCAUCAC-3′, 5′-UGAAGUGGCUUGUGGUGCUGAGUGA-3′ and 5′-CCUACAUCCCGAUCGAUGAUGUUGA-3′ respectively. Human miR-30a and miR-30e mimics, control mimics, and miR-30a inhibitors and control inhibitors were synthesized from RiBoBio (Guangzhou, China). Matrigel was from BD-Biosciences (Bedford, MA, USA). All *in vitro* experiments were performed at least in triplicate, and representative data are presented.

### Cell transfection

FuGENE HD Transfection Reagent (Roche Applied Science, Mannheim, Germany) was used for transfection of pcDNA3.1 or RUNX3/pcDNA3.1 plasmid into AGS, BGC-823 or SGC-7901. Lipofectamine 2000 (Invitrogen) was used to transfect siRNA into BGC-823 or SGC-7901 cells. All transfection procedures followed the protocol of the manufacturer.

### Reporter vector construction and luciferase assay

Luciferase reporter vector pMIR-REPORT (Ambion, Austin, TX, USA) was used to generate luciferase reporter constructs. The 366-bp miR-30a binding sequence at the 3′ untranslated region (3′ UTR) of human vimentin gene (Vim) was amplified and cloned into the SpeI/HindIII sites of a luciferase gene in the pMIR-REPORT luciferase vector (pMIR-Vim/wt). Two miR-30a complementary sites with the sequence GTTTAC in the 3′ UTR were mutated to remove complementarity with miR-30a by use of a QuikChange site–directed mutagenesis kit with pMIR-Vim/wt as the template. All the primer sequences were listed in Table 2. The mutants were named pMIR-Vim/mut1 and pMIR-Vim/mut2. Gastric cancer cells were seeded in 24-well plates and transiently transfected with appropriate reporter plasmid and miRNA by use of Lipofectamine 2000. The cells were harvested and lysed after 48 hrs. Luciferase activity was measured by use of the Dual-Luciferase Reporter Assay System (Promega, Madison, WI, USA). Renilla luciferase was used for normalization. For each plasmid construct, transfection experiments were performed in triplicate.

**Table 2:** Primer sequences for construction of wild-type (pMIR-Vim/wt) and mutants (pMIR-Vim/mut1 and pMIR-Vim/mut2) of the 3′ UTR of vimentin

Category	Primers
Sequences for construction of pMIR-Vim/wt	5′-ACTAGTAAATTGCACACACTCAGTGC-3′ 5′-AAGCTTGAGTTTTTCCAAAGATTTATTG-3′
Sequences for construction of pMIR-Vim/mut1	5′-GATTTAGAAAAAATGCGCTGACATAATCTA-3′ 5′-TAGATTATGTCAGCGCATTTTTTCTAAATC-3′
Sequences for construction of pMIR-Vim/mut2	5′-CAACATAATCTATCGCGACGAAAAATCT-3′ 5′-AGATTTTTCGTCGCGATAGATTATGTTG-3′

### RNA extraction and real-time PCR

Total RNA in cells and tissue was extracted by use of TRIzol reagent (Invitrogen). Primers for miR-30a, miR-30e and U6 were from RiBoBio. Primers for vimentin, E-cadherin, Snail, Slug, Twist and β2-M were listed in Table 3. The first-strand cDNA was synthesized with random primers (N6) or miRNA-specific primers and M-MLV reverse transcriptase (Ferments). Real-time PCR involved the use of the ABI7700 sequence detector (Applied Biosystems, Foster City, CA) with the SYBR Green kit (TakaRa, Dalian, China). Calculation of target mRNA levels was based on the CT method and normalization to human β2-M or U6 expression. All reactions were run in triplicate.

**Table 3:** Primer sequences used in the study

Genes	Primers
Vimentin	5′-CTCTTCCAAACTTTTCCTCCC (forward) 5′-AGTTTCGTTGATAACCTGTCC (reverse)
E-cadherin	5′-TTCCTCCCAATACATCTCCC (forward) 5′-TTGATTTTGTAGTCACCCACC (reverse)
Snail1	5′-GGAAGCCTAACTACAGCGAGCT (forward) 5′-TCCCAGATGAGCATTGGCA (reverse)
Slug	5′-CGCCTCCAAAAAGCCAAAC (forward) 5′-CGGTAGTCCACACAGTGATG (reverse)
Twist1	5′-GTCCGCAGTCTTACGAGGAG (forward) 5′-GCTTGAGGGTCTGAATCTTGCT (reverse)
β2-M:	5′-GAATTGCTATGTGTCTGGGT (forward) 5′-CATCTTCAAACCTCCATGATG (reverse)
miR-30a (ChIP)	5′-CCAGCCAAAGTAGGAAGGAG-3′ (forward) 5′-CCGGAGTTCCTCAGACAAGAG-3′ (reverse)

### Western blot analysis

Total cellular or tissue protein was extracted with the use of RIPA lysis buffer. Protein concentrations were measured by use of the BCA reagent kit (Merck, Darmstadt, Germany). Cellular proteins were resolved by SDS-PAGE and transferred to a nitrocellulose membrane, which was probed with specific primary antibodies against RUNX3 (Abcam, Cambridge, UK), E-cadherin (Cell Signaling Technology, Billerica, MA, USA), vimentin (Cell Signaling), or β-actin (Sigma-Aldrich, St. Louis, MO, USA), then secondary antimouse or rabbit horseradish peroxidase-conjugated IgG (Bio-Rad, Hercules, CA, USA), and developed with the chemiluminescence method (ECL, Millipore, Billerica, MA, USA).

### Microarray analysis

Total RNA was harvested from cells by use of TRIzol (Invitrogen) and the miRNeasy mini kit (Qiagen, Valencia, CA, USA). Samples were labelled by use of the miRCURY LNA microRNA Hy3/Hy5 Power labelling kit and hybridized on the miRCURY LNA Array (v.16.0). After washing steps, the slides were scanned by use of the Axon GenePix 4000B microarray scanner (Molecular Devices, Inc., Sunnyvale, CA, USA). Scanned images were imported into GenePix Pro 6.0 (Axon) for grid alignment and data extraction.

### Matrigel invasion assay

AGS, BGC-823 and SGC-7901 cells transfected with pcDNA3.1 or the RUNX3 overexpression plasmid RUNX3/pcDNA3.1 were harvested and re-suspended in serum-free RPMI-1640 medium, and 1 × 10^5^ cells were seeded into the upper 24-well chambers coated with 150 mg Matrigel in a cell invasion system (BD Biosciences). Medium with 10% FBS was added to the lower chambers as a chemoattractant. After 36 hrs, cells remaining on the upper surface of the membrane were removed with the use of a cotton swab, and cells that had invaded the membrane filter were fixed with 100% methanol, stained in a dye solution containing 0.05% crystal violet and photographed under a microscope. The number of invading cells was counted from three independent experiments.

### Chromatin immunoprecipitation (ChIP)

Chromatin immunoprecipitation assay was as previously described 7. Briefly, AGS cells (no endogenous RUNX3 expression) transfected with pcDNA3.1 or RUNX3/pcDNA3.1 plasmid were cross-linked by incubation in 1% (v/v) formaldehyde-containing medium for 10 min. at 37°C, then sonicated to form soluble chromatin. Antibodies against RUNX3 (Abcam) were used to precipitate DNA fragments bound by their corresponding elements. The protein–DNA complex was collected with the use of protein A Sepharose beads (Millipore), eluted and reverse cross-linked. After treatment with protease K (Qiagen), samples were extracted with phenol/chloroform and precipitated with ethanol. The recovered DNA was re-suspended in TE buffer and amplified by PCR with primers for the miR-30a promoter listed in Table 3.

### Mouse model

Female athymic BALB/c nude mice (Shanghai Slac Laboratory Animal Co., China) were used to analyse metastatic potency *in vivo*. BGC-823 cells were transfected with pcDNA3.1 or RUNX3/pc3.1, then screened with G418 to obtain stably transfected cells. Gastric cancer cells (6 × 10^6^ per mouse) were injected intravenously into the mouse tail vein. Animals were killed after 4 weeks, lungs were excised and tumours on the lung surface were counted. Lungs were fixed in formaldehyde solution and stained with haematoxylin and eosin to examine metastatic nodules.

### Statistical analysis

Data are presented as mean ± SD and compared by two-sided unpaired *t* test. Correlation analyses of RUNX3, miR-30a and vimentin in GC samples were made using linear regression. All experiments were repeated three times. Data analysis involved the use of SigmaStat3.1 (Systat Software, Inc., Richmond, CA, USA). *P* < 0.05 was considered statistically significant.

## Results

### RUNX3 inhibition induced a mesenchymal phenotype in gastric cancer cells and stimulated tumour cell invasion

**Figure 1:**
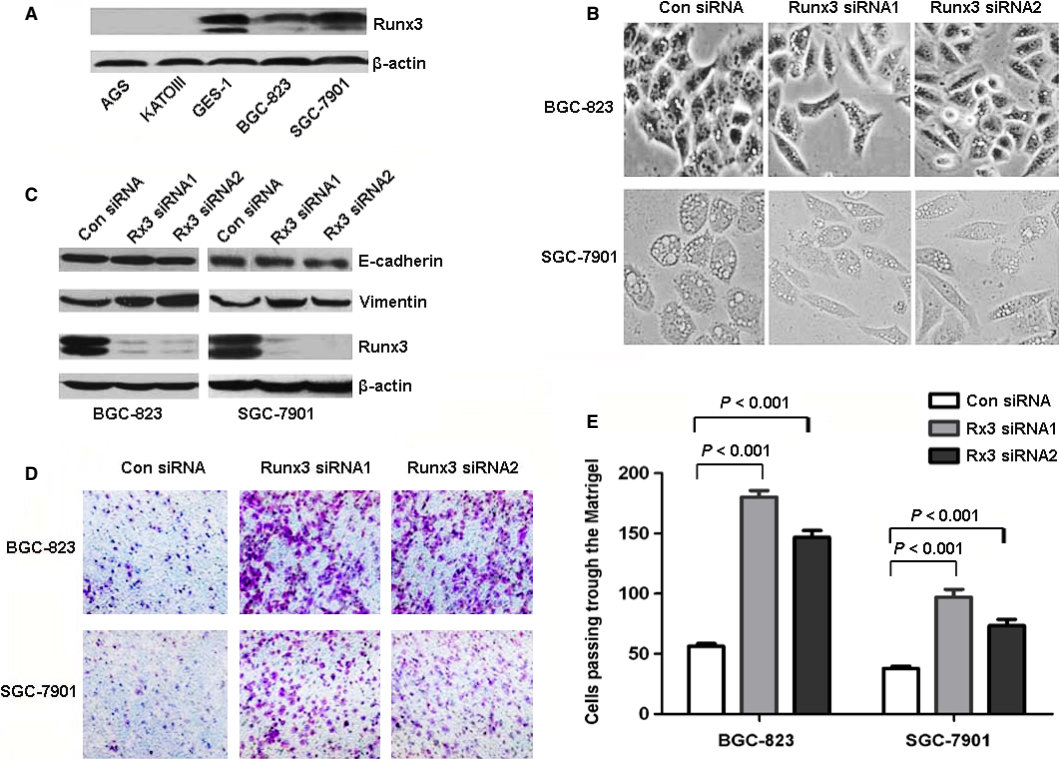
Runt-related transcription factor 3 (RUNX3) inhibition stimulated epithelial–mesenchymal transition (EMT)-like alteration in morphological features and increased vimentin expression and invasion of gastric cancer cells. (A) Western blot analysis of RUNX3 protein level in gastric cancer cell lines and immortalized gastric epithelial cells. (B) EMT-like morphological changes in BGC-823 and SGC-7901 cells with RUNX3 siRNA. Cells were transfected with control siRNA (left panel) or RUNX3 siRNA1 (middle panel) or siRNA2 (right panel) and cellular morphology was examined under light microscopy after 72 hrs. (C) Western blot analyses of protein levels of RUNX3, E-cadherin and vimentin in BGC-823 and SGC-7901 cells transfected with control siRNA or RUNX3 siRNA1 or siRNA2. (D) Matrigel assay of the invasion ability of BGC-823 and SGC-7901 cells transfected with control siRNA or RUNX3 siRNA1 or siRNA2. (E) The number of cells passing through a Matrigel filter in BGC-823 and SGC-7901 cells. Data are mean ± SD from three experiments.

First, we investigated the expression of RUNX3 in gastric cell lines. The human immortalized gastric epithelial cell line GES-1 showed the highest expression of RUNX3; AGS and KATOIII lines show no expression of RUNX3; BGC-823 and SGC-7901 lines showed a moderate expression of RUNX3 (Fig. 1A). The result was consistent with the Guo *et al*. results 33. Therefore, we chose BGC-823 and SGC-7901 cells for RUNX3 siRNA and expression vector transfection and AGS cells for RUNX3 expression vector transfection.

Runt-related transcription factor 3-specific siRNA transfected into BGC-823 and SGC-7901 cells to knock down RUNX3 expression produced an elongated fibroblastoid-like morphologic alteration in transfected cells (Fig. 1B). Therefore, we investigated the expression of the epithelial and mesenchymal markers E-cadherin and vimentin, respectively, in gastric cancer cells transfected with control siRNA and RUNX3 siRNA. Gastric cancer cells transfected with RUNX3 siRNA showed decreased RUNX3 protein expression (Fig. 1C). The expression of E-cadherin was not changed significantly, but that of vimentin was increased in cells with RUNX3 knockdown (Fig. 1C). Because the EMT programme activation contributes to cancer cell invasion, we determined the invasive potential of cells transfected with control siRNA or RUNX3 siRNA. Invasion of BGC-823 and SGC-7901 cells was greater with RUNX3 siRNA than control siRNA (Fig. 1D and E).

### *RUNX3* overexpression inhibited tumour cell invasion and decreased the expression of vimentin in gastric cancer cells

**Figure 2:**
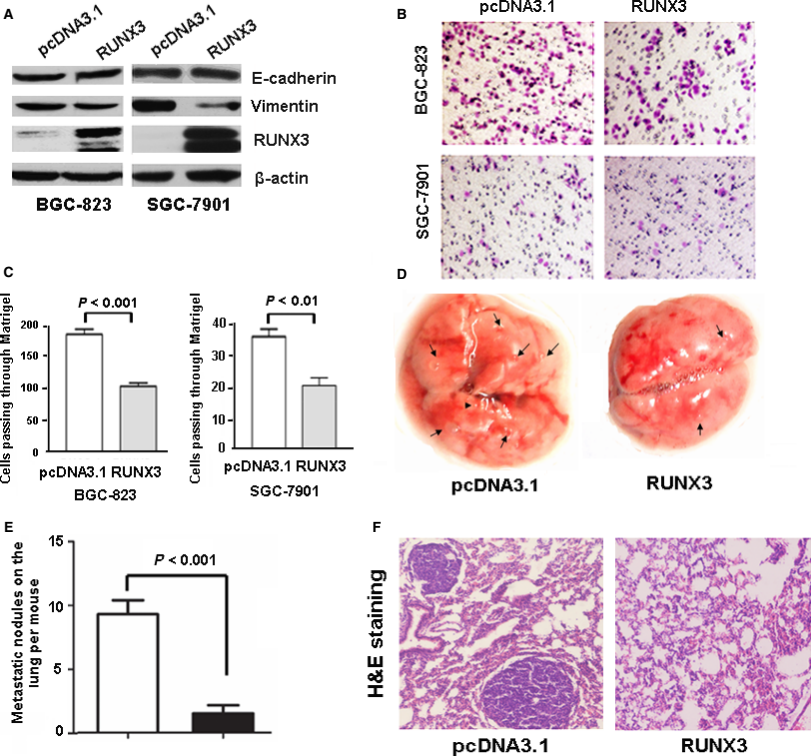
Runt-related transcription factor 3 (RUNX3) overexpression leads to downregulated vimentin expression and diminished invasion and migration ability *in vitro* and *in vivo*. (A) Western blot analyses of protein levels of RUNX3, E-cadherin and vimentin in BGC-823 and SGC-7901 cells transfected with pcDNA3.1 control or RUNX3/pcDNA3.1 (RUNX3) plasmids. (B) Matrigel assay of the invasion of BGC-823 and SGC-7901 cells transfected with pcDNA3.1 or RUNX3/pcDNA3.1. (C) The number of cells passing through a Matrigel filter. Data are mean ± SD from three experiments. (D) Lungs from mice transfected with pcDNA3.1– and RUNX3/pc3.1–BGC-823 cells. (E) Metastatic nodules on mouse lungs. Data are mean ± SD from three experiments. (F) haematoxylin and eosin staining of lung tissues.

We next wondered whether RUNX3 overexpression negatively affected the EMT programme and cell invasion. We transfected pcDNA3.1 or RUNX3/pcDNA3.1 plasmid into BGC-823,SGC-7901 and AGS cells. Cells transfected with RUNX3/pcDNA3.1 showed increased RUNX3 protein expression (Fig. 2A, Figure S1). Runx3 overexpression decreased vimentin protein level (Fig. 2A, Figure S1) and inhibited cell invasion in BGC-823,SGC-7901 and AGS cells (Fig. 2B and C, Figure S1).

### RUNX3 inhibited tumourigenesis and colonization of gastric cancer cells *in vivo*

We then examined whether RUNX3 overexpression inhibited the metastasis of gastric cancer cells *in vivo*. We injected BGC-823 cells transfected with pcDNA3.1 or RUNX3/pc3.1 into the tail vein of nude mice and examined their lungs for tumour seeding. Metastatic nodules were fewer with RUNX3/pc3.1 than pcDNA3.1 transfection (Fig. 2D–F).

### RUNX3 inhibition or overexpression had no effect on vimentin mRNA level

**Figure 3:**
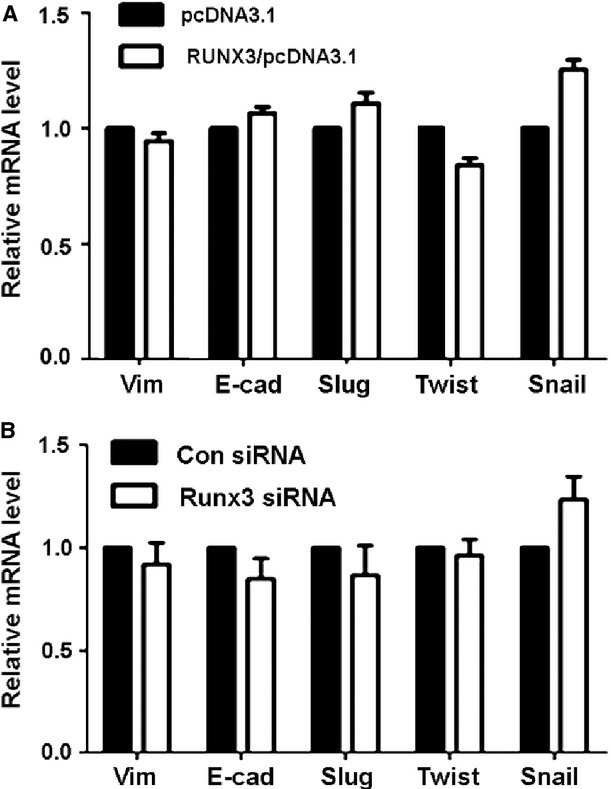
Runt-related transcription factor 3 (RUNX3) overexpression or inhibition had no effect on the transcription of epithelial–mesenchymal transition genes. Quantitative RT-PCR (qRT-PCR) analysis of mRNA levels of vimentin, E-cadherin, Slug, Twist and Snail1 in SGC-7901 cells treated with (A) pcDNA3.1 or RUNX3/pcDNA3.1 plasmids and (B) control or RUNX3 siRNA1.

To further test whether RUNX3 affects the transcription of genes regulating the EMT, we examined the effect of RUNX3 on the mRNA level of vimentin, E-cadherin, Slug, Twist and Snail in BGC-823 and SGC-7901 cells. Neither RUNX3 inhibition nor overexpression had an effect on the mRNA levels of these genes (Fig. 3A and B). Therefore, RUNX3 did not regulate vimentin at the transcriptional level.

**Figure 4:**
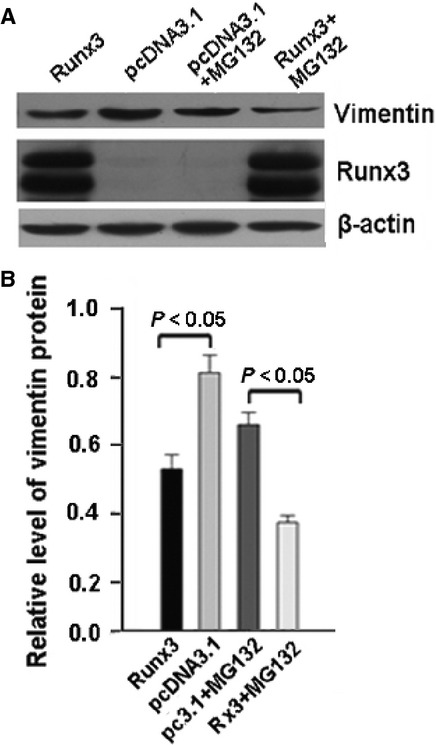
MG132 had no effect on runt-related transcription factor 3 (RUNX3)-induced downregulation of vimentin. SGC-7901 cells were transfected with pcDNA3.1 control or RUNX3/pcDNA3.1 plasmid. At 36 hrs, cells were treated with or not with MG132 (10 μM) for 6 hrs. (A) Western blot analysis of protein level of vimentin and (B) quantification. Data are mean ± SD from three experiments.

We next determined whether the RUNX3-mediated decrease in vimentin resulted from proteasomal degradation. We treated cells transfected with RUNX3 expression vector with the proteasome inhibitor MG132 and detected the protein level of vimentin by western blot analysis. The addition of MG132 did not abolish the ability of ectopically expressed RUNX3 to decrease vimentin protein level in gastric cancer cells (Fig. 4A and B).

### RUNX3 activated miR-30a expression

**Figure 5:**
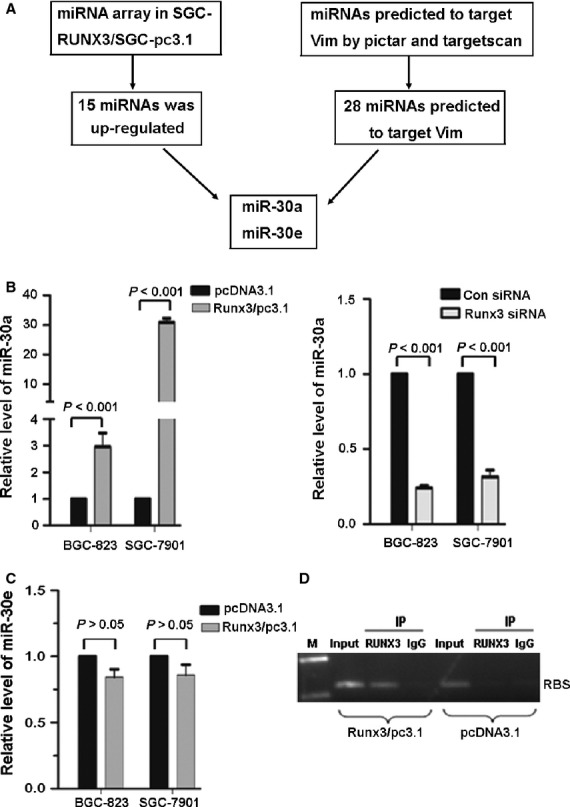
Runt-related transcription factor 3 (RUNX3) increased miR-30a expression in BGC-823 and SGC-7901 cells. (A) Schema for identification of the putative miRNA(s) that could be activated by RUNX3 and partly complementary to a conserved site within the 3′ untranslated region of vimentin. (B and C) qRT-PCR analysis of mRNA levels of miR-30a and miR-30e, respectively, in BGC-823 and SGC-7901 cells transfected with RUNX3 overexpression plasmid or RUNX3 siRNA. Data are mean ± SD from three experiments. (D) ChIP assay for RUNX3 occupancy on the upstream of miR-30a. AGS cells were transfected with RUNX3 expression vectors and ChIP was performed with chromatin derived from the cells.

Because miRNAs can suppress gene expression by binding to the 3′ UTR of target mRNAs and these interactions may inhibit translation of the targeted mRNAs or their degradation, we next determined whether RUNX3 activated an miRNA, then whether the miRNA targets to degrade vimentin. We first used miRNA array to screen miRNAs regulated by RUNX3 and found 15 miRNAs activated by RUNX3. We searched miRNA databases to analyse these differentially expressed miRNAs and found that among the 15 miRNAs, only miR-30a and miR-30e were partly complementary to the conserved site within the 3′ UTR of vimentin (Fig. 5A). Therefore, we overexpressed or knocked down RUNX3 in BGC-823 and SGC-7901 cells and used qRT-PCR to detect the miR-30a and miR-30e mRNA levels in these cells. We found that RUNX3 overexpression up-regulated and inhibition downregulated the expression of miR-30a, but had no effect on miR-30e level (Fig. 5B and C).

To further determine whether RUNX3 regulate the expression of miR-30a by directly binding to the corresponding genomic sequences, we used Genomatrix to analyse the upstream region of mature miR-30a and found a consensus binding sequences of RUNX3 ‘TGTGGT’ (RBS) between the nucleotides -3854 to -3861. As AGS cell has no engeneous RUNX3 expression, we transfected RUNX3/pcDNA3.1 into the cells and used ChIP assay to confirm the direct binding of RUNX3 to the RBS sites in RUNX3-overexpressing AGS cells using anti-Runx3 antibody (Fig. 5D).

### miR-30a targeted the vimentin 3′ UTR directly and downregulated vimentin protein expression

**Figure 6:**
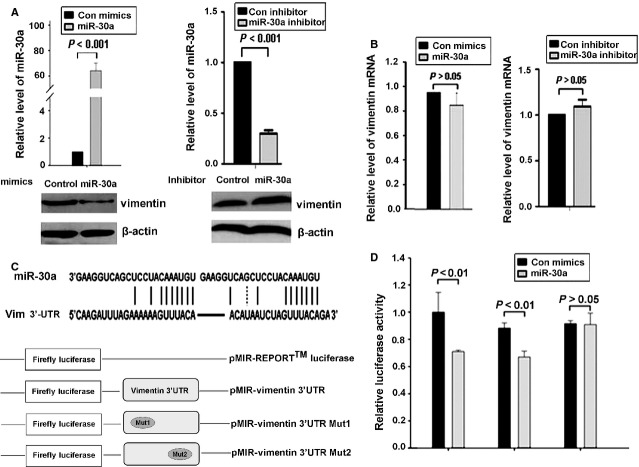
miR-30a directly binds to the 3′ untranslated region (UTR) of vimentin and downregulated vimentin protein expression. (A and B) western blot and qRT-PCR analysis of mRNA and protein levels, respectively, of vimentin in SGC-7901 cells transfected with miR-30a mimics and inhibitor. (C) Upper: two predicted conserved sites of miR-30a in the 3′ UTR of vimentin. Lower: diagram of vimentin 3′ UTR-containing reporter construct. Two mutations were generated at the predicted miR-30a binding sites located in the vimentin 3′ UTR. (D) The wild-type or mutant reporter constructs were cotransfected with miR-30a or control into SGC-7901 cells. Luciferase activity was determined at 48 hrs and was normalized to Renilla luciferase activity.

Next, we investigated whether miR-30a affected the expression of vimentin. We transfected the inhibitor or mimics of miR-30a into SGC-7901 cells and used qRT-PCR and Western blot analyses to detect the expression of vimentin. miR-30a mimics inhibited and miR-30a inhibitor induced vimentin protein level, but had no effect on its mRNA level (Fig. 6A and B). To further determine whether miR-30a directly targets vimentin mRNA in gastric cancer cells, we inserted the full-length 3′ UTR of vimentin into the pMIR-REPORT luciferase vector (pMIR-Vim; Fig. 6C) and investigated the effect of miR-30a on the luciferase activity of pMIR-Vim. miR-30a significantly reduced the activity of the luciferase reporter of pMIR-Vim (Fig. 6D). Because two conserved sites within the 3′ UTR of vimentin were identified to be complementary to miR-30a, we generated two 3′ UTR mutants, pMIR-Vim/mut1 and pMIR-Vim/mut2 (Fig. 6C), and investigated the effect of miR-30a on their luciferase activity. The luciferase activity was significantly reduced in the presence of miR-30a with pMIR-Vim/mut1 but not pMIR-Vim/mut2 (Fig. 6D). Therefore, the second region of the 3′ UTR of vimentin is important for miR-30a binding.

### mir-30a was critical for RUNX3-mediated cell invasion inhibition and vimentin downregulation

**Figure 7:**
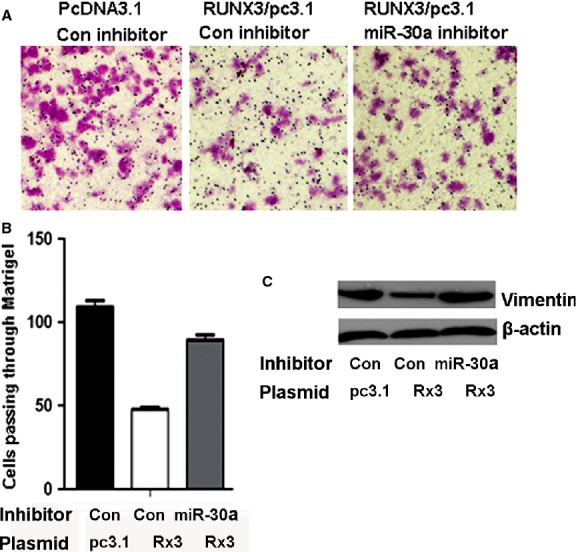
Runt-related transcription factor 3 (RUNX3)-mediated inhibition of cell invasion and downregulation of vimentin depends on miR-30a activation. (A) miR-30a inhibitor abrogated RUNX3-mediated inhibition of cell invasion. Matrigel assay of the invasion ability of SGC-7901 cells with different transfection conditions. (B) The number of cells invading a Matrigel filter in SGC-7901 cells. Data are mean ± SD from three experiments. (C) Western blot analysis of miR-30a inhibitor blocking RUNX3-mediated downregulation of vimentin protein level.

We next ascertained whether RUNX3 regulates EMT and vimentin expression *via* an miR-30a–dependent mechanism. We knocked down miR-30a with an inhibitor of miR-30a in RUNX3-overexpressed gastric cancer cells and detected cell invasion and the expression of vimentin. The miR-30a inhibitor abrogated RUNX3-mediated inhibition of cell invasion (Fig. 7A and B). The RUNX3-mediated downregulation of vimentin protein level was also abrogated with the miR-30a inhibitor (Fig. 7C, Figure S2). Therefore, RUNX3-mediated cell invasion inhibition and vimentin downregulation depended on miR-30a.

### RUNX3 expression was associated with miR-30a and vimentin in primary gastric cancer

**Figure 8:**
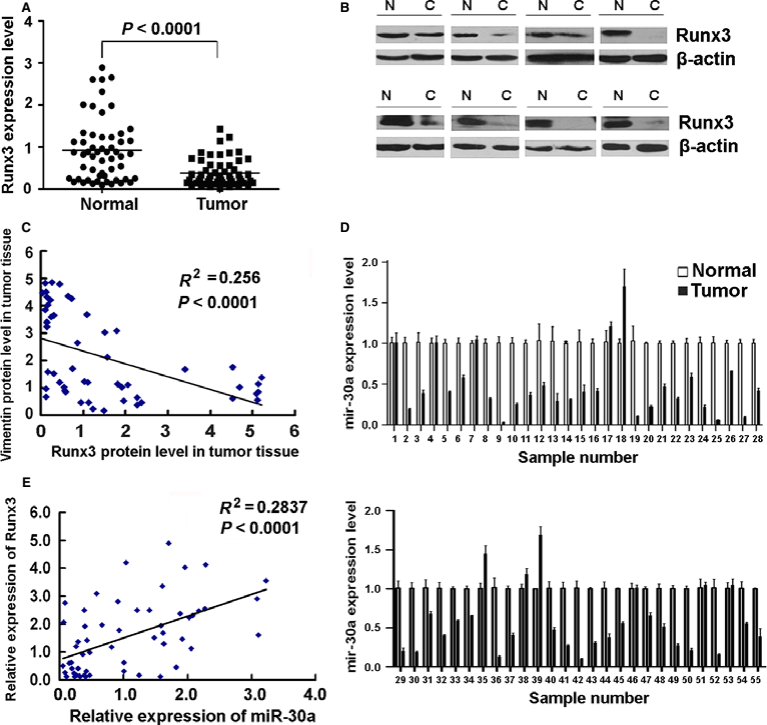
Runt-related transcription factor 3 (RUNX3) expression is associated with miR-30a and vimentin in primary gastric cancer tissues. (A) Western blot analysis of RUNX3 protein level in representative gastric cancer specimens and adjacent normal gastric mucosa. β-actin was a control. N and C, normal and cancer tissues. (B) Relative RUNX3 protein expression levels in gastric cancer tissues and adjacent normal gastric mucosa (RUNX3/GAPDH,*n* = 25, *P* = 0.0011). Horizontal lines represent the mean. (C) qRT-PCR analysis of mRNA level of miR-30a in gastric cancer specimens and adjacent normal gastric mucosa. Data are mean ± SD from three experiments. (D) Regression analysis of correlation of RUNX3 and miR-30a expression. (E) Regression analysis of correlation of RUNX3 and vimentin expression. Each point represents one cancer sample.

Finally, we determined RUNX3 and vimentin expression in 55 primary tumour samples from gastric cancer patients by western blot analysis and miR-30a expression by qRT-PCR analysis. In all, 35/55 (63.6%) of the clinical gastric cancer specimens showed reduced expression of RUNX3 as compared with surrounding normal mucosa. Statistical analysis showed that the expression of RUNX3 in tumour tissue was significantly lower than that in surrounding normal mucosa (Fig. 8A and B). A highly significant negative correlation between RUNX3 and vimentin protein was observed in these gastric cancer samples (*P* < 0.0001; Fig. 8C). The expression of miR-30a was reduced in 43/55 (78.2%) of the gastric cancer samples as compared with the surrounding normal mucosa (Fig. 8D). In all, 29/55 samples showed the same trend of decreased RUNX3 and miR-30a expression in tumour rather than normal tissue. Statistical analysis showed that RUNX3 and miR-30a levels were highly correlated in gastric cancer samples (*P* < 0.0001; Fig. 8E).

## Discussion

Runt-related transcription factor 3 is closely associated with the development and progression of gastric cancer. Clinical studies showed that a decrease or loss of RUNX3 expression was inversely related to survival and was an independent predictor of poor patient outcome. As well, restoration of RUNX3 expression greatly suppresses tumour growth and metastasis 34. However, how RUNX3 is involved in cancer progression remained incompletely understood. In this study, we addressed whether RUNX3 has a role in the EMT, related to cancer relapse and metastasis, in gastric cancer. Knockdown of RUNX3 promoted the EMT and increased the protein expression of the mesenchymal marker vimentin in human gastric cancer cells. Overexpression of RUNX3 suppressed cell invasion and decreased the protein expression of vimentin in gastric cancer cells and inhibited gastric cancer cell colonization in nude mice. Furthermore, overexpression of RUNX3 increased the expression of miR-30a, which directly targeted the 3′ UTR of vimentin and decreased its protein level. An miR-30a inhibitor abrogated RUNX3-mediated inhibition of cell invasion and downregulated vimentin. In gastric cancer patients, levels of RUNX3 were positively correlated with miR-30a and negatively associated with vimentin. Thus, RUNX3 suppressed gastric cancer cell invasion and vimentin expression by activating miR-30a. We provide evidence that RUNX3 plays a significant role in regulating the EMT, thereby contributing to cancer progression.

In this study, RUNX3 inhibition and overexpression increased and decreased human gastric cancer cell invasion respectively. *In vivo*, RUNX3 inhibits human gastric cancer cells metastasis. Tanaka *et al*. 35 also found that RUNX3 reversed the EMT in hepatocellular carcinoma, but the mechanism underlying RUNX3 action is different in gastric cancer cells. Tanaka *et al*. 35 showed that ectopic RUNX3 protein expression induced E-cadherin expression and suppressed vimentin expression in hepatocellular carcinoma cells with low EMT. However, in our study, RUNX3 did not induce E-cadherin in human gastric cancer cells. Different tumour cell types may have different mechanisms in regulating the EMT.

We found that RUNX3 greatly inhibited vimentin protein expression, but had no effect on vimentin transcription. Because the transcription factors Snail, Slug/Snail2 and Twist play an important role in regulating the EMT 36, we determined whether RUNX3 regulates the expression of these transcription factors. Our results showed that RUNX3 did not affect the mRNA level of these factors, so RUNX3 does not regulate the EMT at the transcription level. We further investigated whether RUNX3 regulates vimentin protein expression through a ubiquitin-proteasome degradation pathway. The addition of the proteasome inhibitor MG132 had no effect on RUNX3-mediated downregulation of vimentin protein, so the ubiquitin-proteasome pathway was not involved.

MiRNAs can interact with complementary sequences in the 3′ UTR of the target mRNA to induce translational repression or target degradation 37. Recently, many studies have suggested that miRNAs contribute to the invasion and metastasis of various types of human cancers by regulating the EMT 37–42. Vimentin is one of the targets that can be regulated by miRNAs. Yamasaki *et al*. 42 showed that miR-138 targeted vimentin and inhibited cell migration and invasion in renal cell carcinoma. Cheng *et al*. 38 showed that miR-30a could directly bind to the 3′ UTR of vimentin and inhibit its translation, then reduce the protein level of vimentin in breast cancer. Therefore, we considered that RUNX3 regulates the expression of some miRNAs, which contributes to the downregulation of vimentin protein and the inhibition of invasion and metastasis of gastric cancer. First, we used miRNA array to detect the miRNAs that could be regulated by RUNX3 and detected 15 miRNAs activated by RUNX3. Among these miRNAs, miR-30a and miR-30e were predicted to target to vimentin. We overexpressed or knocked down RUNX3 in gastric cancer and detected the mRNA expression of miR-30a and miR-30e. Only miR-30a was significantly activated by RUNX3. To investigate whether miR-30a regulates the expression of vimentin, we transfected gastric cancer cells with miR-30a mimics and detected the protein and mRNA levels of vimentin. Overexpression of miR-30a decreased the protein level of vimentin in human gastric cancer cells, but had no effect on the mRNA level. In contrast, inhibition of miR-30a increased the protein level of vimentin. As well, miR-30a could directly bind to the 3′ UTR of vimentin, which is consistent with the results of Cheng *et al*. 38 in breast cancer. To further detect whether RUNX3 downregulated vimentin expression through miR-30a, we added the miR-30a inhibitor into gastric cancer cells transfected with the RUNX3 expression vector and found that the miR-30a inhibitor abolished the RUNX3-mediated downregulation of vimentin and the inhibition of gastric cancer cell invasion. Therefore, RUNX3 downregulated vimentin and inhibited gastric cancer cell invasion through an miR-30a–dependent mechanism.

Collectively, our data suggest a novel molecular mechanism for the tumour suppressor activity of RUNX3. Targeting the RUNX3 pathway may help to control gastric cancer invasion and metastasis by inhibiting the EMT.

## Supporting information

Figure S1 RUNX3 overexpression leads to downregulated vimentin expression and diminished invasion ability in AGS cells.

Figure S2 miR-30a inhibitor blocked RUNX3-mediated downregulation of vimentin protein level in AGS cells.

## References

[1] Vogiatzi P, Vindigni C, Roviello F (2007). Deciphering the underlying genetic and epigenetic events leading to gastric carcinogenesis. J Cell Physiol.

[2] DiMario F, Cavallaro LG, Cavestro GM (2006). Are there useful biomarkers for gastric cancer?. Dig Liver Dis.

[3] Nguyen DX, Bos PD, Massague J (2009). Metastasis: from dissemination to organ-specific colonization. Nat Rev Cancer.

[4] Fidler IJ (2003). The pathogenesis of cancer metastasis: the ‘seed and soil’ hypothesis revisited. Nat Rev Cancer.

[5] Chaffer CL, Weinberg RA (2011). A perspective on cancer cell metastasis. Science.

[6] Peinado H, Olmeda D, Cano A (2007). Snail, Zeb and bHLH factors in tumour progression: an alliance against the epithelial phenotype?. Nat Rev Cancer.

[7] Liu Z, Li Q, Li K (2013). Telomerase reverse transcriptase promotes epithelial- mesenchymal transition and stem cell-like traits in cancer cells. Oncogene.

[8] Zhao X, Dou W, He L (2013). MicroRNA-7 functions as an anti-metastatic microRNA in gastric cancer by targeting insulin-like growth factor-1 receptor. Oncogene.

[9] Thiery JP (2002). Epithelial-mesenchymal transitions in tumour progression. Nat Rev Cancer.

[10] Thiery JP, Sleeman JP (2006). Complex networks orchestrate epithelial-mesenchymal transitions. Nat Rev Mol Cell Biol.

[11] Kalluri R, Weinberg RA (2009). The basics of epithelial-mesenchymal transition. J Clin Invest.

[12] Polyak K, Weinberg RA (2009). Transitions between epithelial and mesenchymal states: acquisition of malignant and stem cell traits. Nat Rev Cancer.

[13] Thiery JP, Acloque H, Huang RY (2009). Epithelial-mesenchymal transitions in development and disease. Cell.

[14] Tsuji T, Ibaragi S, Hu GF (2009). Epithelial-mesenchymal transition and cell cooperativity in metastasis. Cancer Res.

[15] Van ZF, Zulehner G, Petz M (2009). Epithelial-mesenchymal transition in hepatocellular carcinoma. Future Oncol.

[16] Zeisberg M, Neilson EG (2009). Biomarkers for epithelial-mesenchymal transitions. J Clin Invest.

[17] Kania MA, Bonner AS, Duffy JB (1990). The Drosophila segmentation gene runt encodes a novel nuclear regulatory protein that is also expressed in the developing nervous system. Genes Dev.

[18] Daga A, Karlovich CA, Dumstrei K (1996). Patterning of cells in the Drosophila eye by Lozenge, which shares homologous domains with AML1. Genes Dev.

[19] Ito Y (1999). Molecular basis of tissue-specific gene expression mediated by the runt domain transcription factor PEBP2/CBF. Genes Cells.

[20] Lin FC, Liu YP, Lai CH (2012). RUNX3-mediated transcriptional inhibition of Akt suppresses tumorigenesis of human gastric cancer cells. Oncogene.

[21] Levanon D, Negreanu V, Bernstein Y (1994). AML1, AML2, and AML3, the human members of the runt domain gene-family: cDNA structure, expression, and chromosomal localization. Genomics.

[22] Otto F, Lubbert M, Stock M (2003). Upstream and downstream targets of RUNX proteins. J Cell Biochem.

[23] Ito Y (2004). Oncogenic potential of the RUNX gene family: ‘overview’. Oncogene.

[24] Li QL, Ito K, Sakakura C (2002). Causal relationship between the loss of RUNX3 expression and gastric cancer. Cell.

[25] Ito K (2011). RUNX3 in oncogenic and anti-oncogenic signaling in gastrointestinal cancers. J Cell Biochem.

[26] Chi XZ, Yang JO, Lee KY (2005). RUNX3 suppresses gastric epithelial cell growth by inducing p21(WAF1/Cip1) expression in cooperation with transforming growth factor {beta}-activated SMAD. Mol Cell Biol.

[27] Yano T, Ito K, Fukamachi H (2006). The RUNX3 tumor suppressor upregulates Bim in gastric epithelial cells undergoing transforming growth factor beta-induced apoptosis. Mol Cell Biol.

[28] Ito K, Chuang LS, Ito T (2011). Loss of Runx3 is a key event in inducing precancerous state of the stomach. Gastroenterology.

[29] Sakakura C, Hasegawa K, Miyagawa K (2005). Possible involvement of RUNX3 silencing in the peritoneal metastases of gastric cancers. Clin Cancer Res.

[30] Peng Z, Wei D, Wang L (2006). RUNX3 inhibits the expression of vascular endothelial growth factor and reduces the angiogenesis, growth, and metastasis of human gastric cancer. Clin Cancer Res.

[31] Chang TL, Ito K, Ko TK (2010). Claudin-1 has tumor suppressive activity and is a direct target of RUNX3 in gastric epithelial cells. Gastroenterology.

[32] Chen Y, Wei X, Guo C (2011). Runx3 suppresses gastric cancer metastasis through inactivation of MMP9 by upregulation of TIMP-1. Int J Cancer.

[33] Guo C, Ding J, Yao L (2005). Tumor suppressor gene Runx3 sensitizes gastric cancer cells to chemotherapeutic drugs by downregulating Bcl-2, MDR-1 and MRP-1. Int J Cancer.

[34] Wei D, Gong W, Oh SC (2005). Loss of RUNX3 expression significantly affects the clinical outcome of gastric cancer patients and its restoration causes drastic suppression of tumor growth and metastasis. Cancer Res.

[35] Tanaka S, Shiraha H, Nakanishi Y (2012). Runt-related transcription factor 3 reverses epithelial-mesenchymal transition in hepatocellular carcinoma. Int J Cancer.

[36] Yang J, Weinberg RA (2008). Epithelial–mesenchymal transition: at the crossroads of development and tumor metastasis. Dev Cell.

[37] Kumarswamy R, Mudduluru G, Ceppi P (2012). MicroRNA-30a inhibits epithelial-to -mesenchymal transition by targeting Snai1 and is downregulated in non-small cell lung cancer. Int J Cancer.

[38] Cheng CW, Wang HW, Chang CW (2012). MicroRNA-30a inhibits cell migration and invasion by down regula- ting vimentin expression and is a potential prognostic marker in breast cancer. Breast Cancer Res Treat.

[39] Chang CJ, Chao CH, Xia W (2011). p53 regulates epithelial-mesenchymal transition and stem cell properties through modulating miRNAs. Nat Cell Biol.

[40] Kim T, Veronese A, Pichiorri F (2011). p53 regulates epithelial-mesenchymal transition through microRNAs targeting ZEB1 and ZEB2. J Exp Med.

[41] Sun L, Yao Y, Liu B (2012). MiR-200b and miR-15b regulate chemotherapy-induced epithelial- mesenchymal transition in human tongue cancer cells by targeting BMI1. Oncogene.

[42] Yamasaki T, Seki N, Yamada Y (2012). Tumor suppressive microRNA-138 contributes to cell migration and invasion through its targeting of vimentin in renal cell carcinoma. Int J Oncol.

