# Supplementary figures and images for: RUNX3 regulates vimentin expression via miR-30a during epithelial–mesenchymal transition in gastric cancer cells
Source: J Cell Mol Med. 2014 Jan 22;18(4):610–23. doi: 10.1111/jcmm.12209 (PMC4000113; doi:10.1111/jcmm.12209)

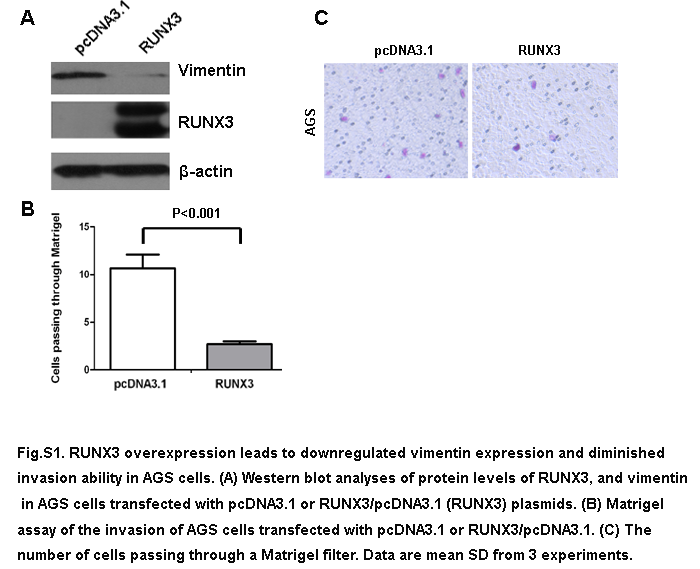

Supplement: Figure S1 [file jcmm0018-0610-sd1.tif]

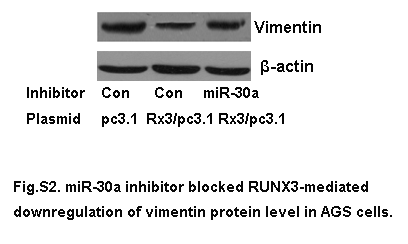

Supplement: Figure S2 [file jcmm0018-0610-sd2.tif]
